# An active compression bandage based on shape memory alloys: a preliminary investigation

**DOI:** 10.1186/1475-925X-13-135

**Published:** 2014-09-11

**Authors:** Hadi Moein, Carlo Menon

**Affiliations:** MENRVA Group, School of Engineering Science, Faculty of Applied Science, Simon Fraser University, 8888 University Drive, Burnaby, BC V5A 1S6 Canada

**Keywords:** Shape memory alloys, Active compression bandage, Orthostatic intolerance

## Abstract

**Background:**

Disorders associated with excessive swelling of the lower extremities are common. They can be associated with pain, varicose veins, reduced blood pressure when standing and may cause syncope or fainting. The common physical remedy to these disorders is the use of compression stockings and pneumatic compression leg massagers, which both attempt to limit blood pooling and capillary filtration in the lower limbs. However, compression stockings provide a constant pressure, and their efficiency has been challenged according to some recent studies. Air compression leg massagers on the other hand, restricts patient mobility. In this work we therefore present an innovative active compression bandage based on the use of a smart materials technology that could produce intermittent active pressure to mitigate the symptoms of lower extremity disorders.

**Methods:**

An active compression bandage (ACB), actuated by shape memory alloy (SMA) wires, was designed and prototyped. The ACB was wrapped around a calf model to apply an initial pressure comparable to the one exerted by commercial compression stockings. The ACB was controlled to apply different values of compression. A data acquisition board and a LabVIEW program were used to acquire both the pressure data exerted by the ACB and the electrical current required to actuate the SMA wires. An analytical model of the ACB based on a SMA constitutive model was developed. An optimizer was implemented to identify optimal parameters of the model to best estimate the performance of the ACB.

**Results:**

The maximum increase in pressure due to the SMA wires activation was 40.8% higher than the initially applied pressure to the calf model. The analytical model of the ACB estimated the behaviour of the ACB with less than 0.32 *mmHg* difference with the experimental results.

**Conclusions:**

The prototyped ACB was able to apply an initial compression comparable to the one applied by commercial compression stockings. Activation of the ACB resulted in an increase of compression up to 9.06 *mmHg*. Comparison between analytical and experimental results showed the analytical model was suitable to predict the behaviour of the ACB.

## Introduction

Gravitational forces affect venous return thus resulting in the accumulation of blood in the lower extremities when standing upright. Considering venous compliance and expansion of the veins with blood, most of the blood volume change occurs in the veins [1]. This leads to an increase of venous volume and pressure in the feet and lower limbs. Venous pooling and enhanced capillary filtration in the upright position may cause most syncopal events to be triggered by orthostatic stress [2]. Syncope, or fainting, is a transient loss of consciousness and postural tone that typically occurs when upright and is correlated with reduced cerebral blood flow [2, 3]. In order to prevent decrease of blood pressure and cerebral blood flow, the reduction of venous return should be compensated - applying an external compression to lower limbs could be effective [4] to assist venus return. One of the current remedies to prevent or delay syncope is the use of compression stockings. This is recommended for those suffering from recurrent orthostatic intolerance, according to the explanation that external counter-pressure of the lower extremities or abdomen can decrease venous pooling and capillary filtration, thereby increasing venous return [5, 6]. This compression could also help the calf muscle pump resulting in reduction of oedema, microcirculation improvement and skin breakdown prevention [7, 8]. Some other studies have also investigated the effectiveness of compression stockings [9–13]. However, their efficiency has been challenged in many studies [11, 12]. One of the limitations of using the compression stockings is the compliance that is necessary to optimize effectiveness [13]. These studies have suggested an individualized tailored design when considering treatment with compression stockings. Furthermore, using of intermittent pneumatic compression has been discussed in the literature [14]. Some researches substantiate the idea that intermittent calf compression could be more effective and should be considered for some group of patients [15, 16]. But its use showed some practical problems such as restriction of patient mobility. The above-mentioned issues of the currently available solutions provide evidence of the need for a novel technology. This technology should provide desired compliance for the patients, be able to apply different ranges of compression based on patients’ leg size and hemodynamic characteristics and alter its amount.

In this work, we proposed the use of shape memory alloys (SMAs) for the design of an ambulatory active compression bandage (ACB). This ACB, which is capable of actively applying an altering external pressure on the lower limbs, has been prototyped using smart alloys technology and its performance was investigated.

SMAs are a group of metallic alloys that exhibit the characteristics of either large recoverable strains or large force due to temperature and/or load changes. They have drawn strong interest in recent years in a large variety of applications including in biomedical engineering. A good summary of design feasibility and applications, as well as overview of SMAs, are presented in [17].

One of the shape memory effect characteristics in SMAs is recovery stress effect. This effect occurs when an elongated SMA sample is restrained while being heated, a significant stress can be produced to prevent the SMA sample from recovering to its original shape [18]. Very few experimental tests or analyses of the behaviour of SMA wires under recovery stress were reported. A recent study has conducted a series of tests of NiTiNb martensitic SMA wires under recovery stress with varying degrees of pre-strain on the wires, and compared the behaviour under recovery stress with that under pre-stressing of the wires. The results showed that the remaining stress (initial applied stress) was reduced by the procedure of loading and unloading additional strain. The more additional strains resulted in the more reduction of remaining stresses [19]. In this study, NiTi SMA wires were utilized in a condition considering recovery stress characteristic of SMAs. This condition makes applying initial constant pressure and then variable increase in pressure to the calf available.

In order to model the phase transformation and thermochemical behaviour of SMAs, extensive research has been conducted. Presented models were mostly one-dimensional. These models were divided into four categories by Brinson *et al.* and included phenomenological laws based on uniaxial stress–strain–temperature data [20]. They were each aimed at describing a different aspect of shape memory behaviour and addressed the thermomechanical behaviour of SMAs based on the phase transformation process on different scales [20]. For this paper, an analytical model was developed to model the behaviour of the ACB. In the model, a well established phenomenological model for describing the behaviour of the SMA wires under thermomechanical loads was used. So the performance of the presented constitutive model could be evaluated for use in further parametric analysis and design improvement of the ACB, utilizing recovery stress characteristics of the SMAs. Genetic algorithm (GA) was utilized to find the key parameters of the analytical model to cover the complete range of applied electrical current in the experiments.

The organization of this paper is as follows. In following section, design and fabrication of the ACB is presented. Then, modelling of the ACB including the constitutive model of SMA is discussed. Next, the experimental setup is described. Finally, the experimental and modelling results are reported and discussed in detail. These sections are followed by the conclusion of the study.

## Design and fabrication of the ACB

**Figure 1:**
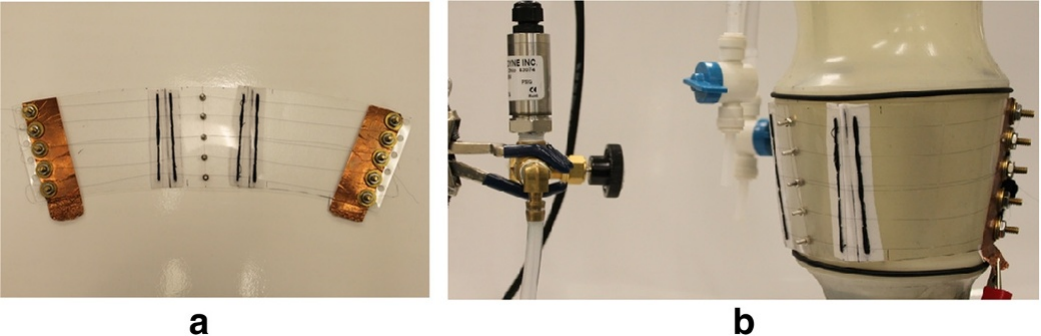
**The active compression bandage (ACB). (a)** The ACB **(b)** wrapped around the CM.

The ACB mainly consists of the SMA wires and a thin plastic sheet. The low temperature Flexinol actuator wire with 102 *μm* diameter was used. A sheet of thermoplastic polycarbonate, known by the trademark name Lexan, with a 0.6 mm thickness was selected to apply the produced pressure by SMA wires to the calf model (CM). As illustrated in Figure 1(a), the fabricated ACB also includes brass washers, screws and nuts to clamp the wires, copper tape to apply the voltage, and small nuts and screws to position them. Furthermore, shoelace was used to fasten the ACB, which has negligible strain when it is in tension.

**Figure 2:**
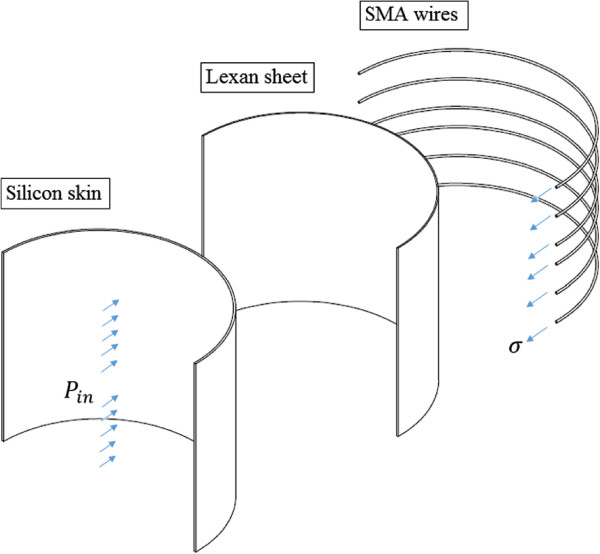
**Schematic exploded section view of the calf model (CM) with the wrapped active compression bandage (ACB).**

The test set up proposed by Pourazadi *et al.* [21] was used to measure the pressure exerted by the ACB. This set-up included the CM and one pressure transducer connected to the CM through tubing, Figure 1(b). It is discussed in the Experimental setup section in detail. To calculate the number of SMA wires in the ACB, it was assumed that the CM had average radius of *r*=5.72 *cm* when the pressure inside it was 9.91 *mmHg*. A schematic exploded section view of the CM with wrapped ACB is shown in Figure 2. In this case, for a single wire we have
1

where *r* is the average radius of the CM, and *P*_*SMA*_ is the circumferential pressure on a SMA wire, *σ* is the longitudinal stress of it and *d* is its diameter. For the ACB, the relationship between pressure inside the chamber and circumferential pressure of wires can be written as
2

where *N* is the number of wires for a specific longitudinal stress in each wire, *P*_*in*_ is the pressure inside the chamber and *w* is the width of the bandage. Equation (2) can be rewritten as
3

Substituting equation (1) in equation (3), we get
4

In this study, the ACB was designed to create a compression range similar to the mildest grade of compression stockings. These stockings apply a pressure of 15−20 *mmHg* (2000−2670 *Pa*) from the knee to the ankle [22]. To fabricate the ACB prototype, the number of the SMA wires was calculated as *N*=6 using equation (4) and assuming 20 *mmHg* of initial compression and the 300 *MPa* for the longitudinal stress of the wires. These values were chosen to generate pressure in the range of commercial stockings and get to the maximum potential of the SMA wires used in the ACB.

## Modelling of the ACB

An analytical model using a constitutive model for the SMA was developed to evaluate the behaviour of the ACB. Inputs of the model are the physical and thermal properties of the SMA wires and the plastic sheet used in the ACB, initial pressure of the chamber, input electrical current, and ambient temperature. The output of the model is the CM pressure that was compared with the experimental data.

The final pressure of the chamber, *P*_*in*_, can be calculated using equation (4). As mentioned above, the number of the SMA wires, *N*, used in the experiments was 6 and the longitudinal stress of a single wire, *σ*, was calculated by the constitutive model.

### Constitutive model of SMA

A well-established consistitive model was proposed by Tanaka *et al.* [23] and later completed by Liang *et al.* [24] and Brinson [25]. Such a model, which was used in several recent works [26–28], was considered in this study to model the thermomechanical behaviour of the SMA wires. The constitutive model shows the relationship between stress rate , strain rate , and temperature rate . The basic equation [24] is
5

where it is assumed [27],
6

in which *D*_*a*_ and *D*_*m*_ are the austenite and martensite Young’s moduli respectively,
7

where *ε*_*L*_ is the maximum recoverable strain and *Θ*_*T*_ is the thermal expansion factor [26]. The martensite fraction (*ξ*) characterizes the extent of the martensite phase transformation. It is a parameter given by 0≤*ξ*≤1. When *ξ*=1, it means that the SMA is completely in the martensite phase, and when *ξ*=0, the SMA wire is completely in the austenite phase [18].

The temperature rate  can be computed by considering the heat transfer equation, which for a SMA wire consists of electrical heating (Joule), heat conduction in the Lexan sheet, and natural convection [26, 29]:
8

where  is mass per unit length of wire, *ρ* is density of wire, *d*=102 *μm* is diameter of wire,  is the circumferential area of the unit length of wire,  the effective conduction area of the unit length of wire, *L* and *K*_*Lexan*_ are the thickness and thermal conductivity of Lexan sheet respectively, and was used in the ACB, *c*_*p*_ is the specific heat, *I* is tha electrical current, *R* is the resistance per unit length of wire, *T* is the temperature of the wire, *T*_*∞*_ is the ambient temperature, and *h*_*c*_ is the heat convection coefficient.

Equation (8) can be rewritten as
9

in which *α*=*R*/*mc*_*p*_ and *β*=[*h*_*c*_*A*_*c*_+(*L*/(*K*_*Lexan*_*A*_*sheet*_)+1/(*h*_*c*_*A*_*sheet*_)]/*mc*_*p*_. Steady state temperature can be calculated using equation (9) and initial temperature.

Time-independent model rate form of the constitutive equation could be obtained by integration of equation (5) respect to time as following [24]
10

where *σ*^*r*^ is the recovery stress and the variables with “0” subscript refer to the initial conditions. As mentioned before, the recovery stress or restrained effect of the SMAs was utilized in this study. The strain rate  was therefore assumed to be equal to zero in this work. Hence the constitutive equation becomes
11

The reverse transformation equation describing the phase transformation from martensite to austenite (heating) [18] is
12

where ,  and *C*_*A*_ are curve-fitting parameters [18]. *A*_*s*_ and *A*_*f*_ are the austenite start and final temperatures respectively. The phase transformation occurs above *A*_*s*_ up to *A*_*f*_. Generally, if there is some stress, the austenite start and final temperatures will be increased [18]. The new austenite start and final temperatures are defined as  and  respectively.

From *T*_0_ to *A*_*s*_, there is no phase transformation, so the recovery stress-temperature relation is linear as following
13

In our study the  is not zero at *T*_0_. The  can be calculated using equations (12) and (13) at the transition point where . It will be as following
14

So the corresponding stress  will be
15

In the temperatures higher than , there is new austenite and the equation (11) will be the constitutive equation.

Using equation (11) and (12) yields the final constitutive equation for the restrained recovery as [24]
16

For above mentioned relation between stress-temperature, for a given temperature, iteration is needed to converge. Above the temperature , there will not be any stress induced for the martensite to austenite transformation. The recovery relation will be linear as following
17

where *T*_0_ is  and  is the recovery stress at  [24]. It results in
18

 can be solved using equations (12) and (18) as
19

In the developed model, an optimization using the GA [30, 31] was conducted to identify optimal values for the parameters *Θ*_*T*_, *ξ*_0_, *A*_*s*_, *A*_*f*_, *C*_*A*_ and *h*_*c*_. The optimized parameters were selected to obtain the model that predicted the experimental results with different applied electrical currents. It is discussed in the Results and discussions section. All parameters used in the calculations are presented in Table 1.

**Table 1:** **Modelling parameters**

Parameter	Description	Unit	Value	Reference
*m*	SMA wire’s mass per unit length	*kg*	5.270×10^−5^	[32]
*A*_*c*_	SMA wire’s circumferential area per unit length	*m* ^2^	2.403×10^−4^	[32]
*A*_*sheet*_	Effective conduction area per unit length	*m* ^2^	7.212×10^−5^	Estimation
*K*_*Lexan*_	Thermal conductivity of Lexan sheet	*W* *m* ^−1^ °*C* ^−1^	0.1947	Data sheet
*C*_*p*_	Specific heat of wire	*J kg* ^−1^ °*C* ^−1^	837	[32]
*R*	SMA wire’s resistance per unit length	*Ω* *m* ^−1^	126	[32]
*T*_*∞*_	Ambient temperature	°*C*	22	Measurement
*T*_0_	Initial temperature	°*C*	22	Measurement
*h*_*c*_	Heat convection coefficient	*W* *m* ^−2^ °*C* ^−1^	164.9	Optimization
*E*_*A*_	Austenite Young modulus	*GPa*	75.0	[26, 27]
*E*_*M*_	Martensite Young modulus	*GPa*	28.0	[26, 27]
*Θ*_*T*_	SMA wire’s thermal expansion factor	*MPa* °*C* ^−1^	0.296	Optimization
*ε*_*L*_	Maximum recoverable strain		0.04	[26, 27]
*ξ*_0_	Initial martensite fraction factor		0.0415	Optimization
*σ*_0_	Average initial stress	*MPa*	305.3	Calculation
*A*_*s*_	Austenite start temperature	°*C*	40.8	Optimization
*A*_*f*_	Austenite final temperature	°*C*	93.6	Optimization
*C*_*A*_	Austenite curve fitting parameter	*MPa* °*C* ^−1^	15.1	Optimization

## Experimental setup

The experimental setup included the calf model (CM) and one pressure transducer connected to the CM through tubing. The CM was designed in three chambers representing the ankle, mid-calf and knee sections of the calf. The chambers can be separated to study a specific region of the calf or can be connected together to study the calf as a whole segment [21]. It was assumed that the human calf is conical and the diameters in the CM were designated based on the reported average size of human calf [33, 34]. Figure 1(b) shows middle section of the CM.

The CM consists of two columns of structure, representing the tibia and fibula bones. The chamber of the CM is covered by a flexible silicone elastomer. Any small variation of the external pressure by the bandage can be detected by using a pressure transducer connected to the enclosed volume of each chamber. A data acquisition board and a LabVIEW program were used to acquire and record the data from the pressure transducer during the experiments. This measured pressure is the summation of the pressure applied by the ACB and the skin pressure. So, prior to the bandage wrapping, the pressure due to the skin itself should be measured and subtracted from the total pressure in the tests results. The chambers were filled in with an incompressible fluid, deionized (DI) water, to limit volume change effects seen for a compressible medium, and the volume of each chamber was kept constant during the experiments. The applied voltage to the SMA wires and produced electrical current were also acquired using the aforementioned data acquisition board and the LabVIEW program simultaneously with the pressure.

The pressure exerted by the ACB consists of two components. First, the *mechanical pressure*, which is the intial pressure produced by having the ACB wrapped around the leg. This produces the initial stress in the SMA wires. Second, the *actuation pressure* that is the result of the heating up of the SMA wires by applying voltage. The total pressure exerted by the ACB on the calf is determined by adding the actuation pressure to the mechanical pressure.

## Results and discussions

Measurements of the compression exerted by the fabricated ACB were recorded on the middle chamber of the CM. The middle chamber was filled in with DI water until the shape of it became conical. The value of the pressure applied by the skin was read prior to the wrapping of the bandage and subtracted from the total pressure.

**Figure 3:**
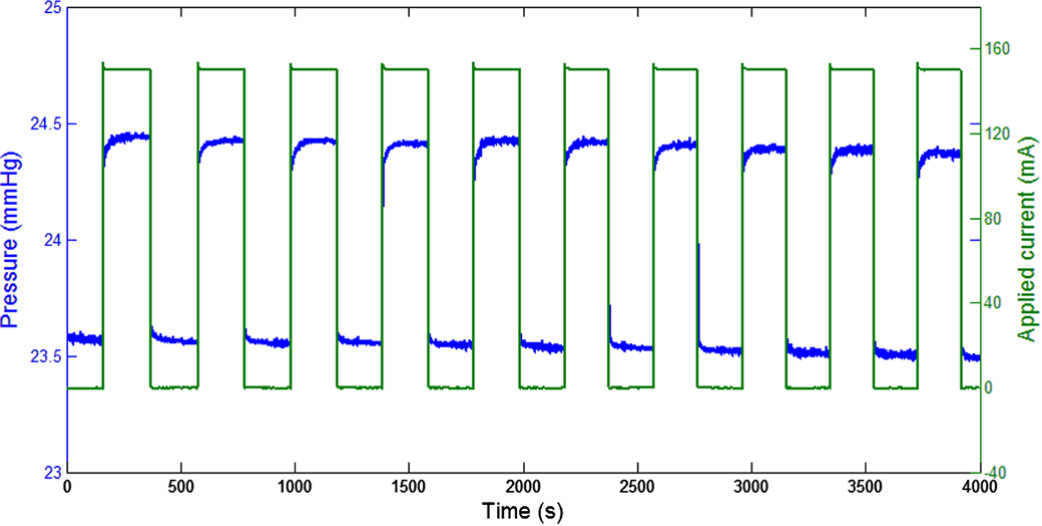
**Cyclic response of the actuaction pressure for 150.3 mA applied current.**

The cyclic response of the actuation pressure for a specific amount of applied electrical current is presented in Figure 3. This figure presents variations of the current applied to each SMA wire and resulting pressure as a function of time. The 10 cycles presented in Figure 3 were obtained after few transient cycles required by the system to yield a repeatable behaviour. In the experiment, the initial mechanical pressure was 23.57 *mmHg* and the average applied electrical current was 150.3 *mA*. Figure 3 shows that a step increase of the current yielded an increase in pressure as a consequence of the contraction of the SMA wires. The behavior of the system did not change during the cycles. Specifically, the minimum pressure, equivalent to the initial mechanical pressure applied by the system when no current was applied, remained constant. This result shows that the plastic strain in the SMA wires was negligible when 150.3 *mA* current was applied.

**Figure 4:**
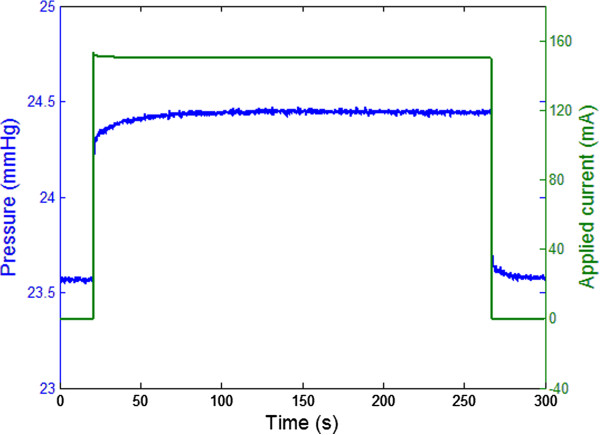
**Activation and deactivation of the shape memory alloy (SMA) wires for 150.3 mA applied current.**

Figure 4 shows a close-up view of a single cycle. It should be noted that this test was part of a different cycling test performed using the same equipment and procedures. By comparing Figures 3 and 4 it can be seen that the behavior of the system was repeatable also between different tests. The close-up view of Figure 4 clearly shows the dynamic behavior of the pressure when current was applied and removed. Specifically, it took 19.25 *s* for the pressure to reach 95*%* of its maximum value when current was applied. Interestingly, it took only 0.94 *s* for the pressure to reach 80*%* of its maximum value. A controller could therefore potentially achieve a fast transient response by suitably controlling the current applied to the system. In the deactivation phase, 80*%* of the minimum pressure was reached in 0.82 *s*. This result indicates that the wires cooled down faster than they heated up.

**Figure 5:**
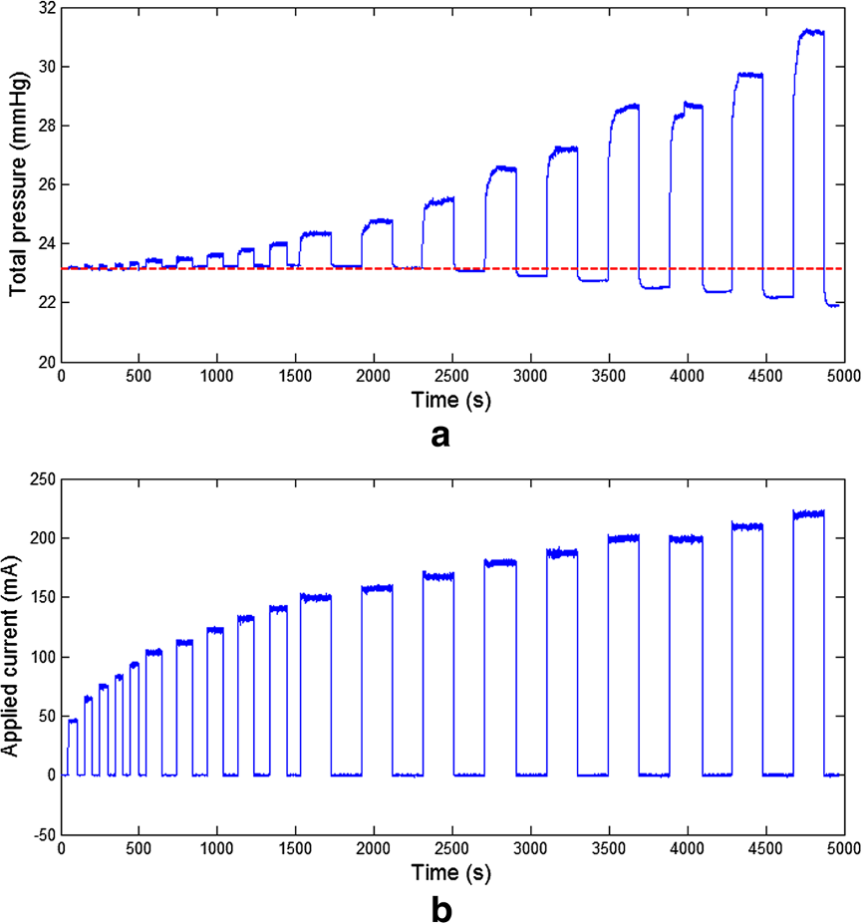
**Actuation of the ACB. (a)** Change of pressure in the chamber and **(b)** applied electrical current to each wire by time.

A different experiment was performed to study how the steady-state pressure changes for different values of current. Ideally, for each variation of current, multiple cycles should be performed as shown in Figure 3. However, given the fact that at high currents the performance of the SMA wires change over time [35], it was decided to gradually increase the current from one cycle to the next. The result of this experiment is illustrated in Figure 5. It should be noted that obtaining the same initial pressure for different tests is currently a practical challenge given the procedure is done manually. This is the reason for which experiments shown in Figures 3, 4 and 5 have slightly different initial pressures. In Figure 5, the initial pressure was 23.16 *mmHg*. This figure shows the change of the pressure in the chamber and the electrical current applied to each wire as a function of time. As expected, the pressure increased with the current. In each cycle, the pressure became stable after few seconds. The dashed red line in Figure 5(a) shows the initial mechanical pressure of the chamber of the CM before the first actuation. It can be observed that the blue line starts crossing the red line at each cycle after approximately 2500 *s*, that is the mechanical pressure at 0 *mA* decreases after a current above a certain threshold is applied (in this case 157.5 *mA*, see Figure 5(b)). Specifically, the higher the current, the lower the mechanical pressure at 0 *mA*. This behaviour can be attributed to a decrease of the longitudinal stress of the SMA wires in their extended configuration. When a high electrical current is applied, a high recovery stress is produced that leads to slip deformation resulting in a plastic strain of the SMA wires. The slip behaviour is an irreversible deformation of a metallic material induced by the movement of the dislocations through the crystal [36]. It is also worth mentioning that under cyclic loading, the NiTi alloys exhibit gradual degradation of pseudoelasticity with cycles, which has been attributed to slip deformation [37].

Results presented in Figure 5 were used to model the system. The above-mentioned decrease in the initial longitudinal stress of the SMA wires was considered in the performed optimization to obtain an accurate model. Specifically, the initial stress, , was calculated using both equation (4) and the experimental measurement of the inner pressure, *P*_*in*_, for each actuation cycle.

**Figure 6:**
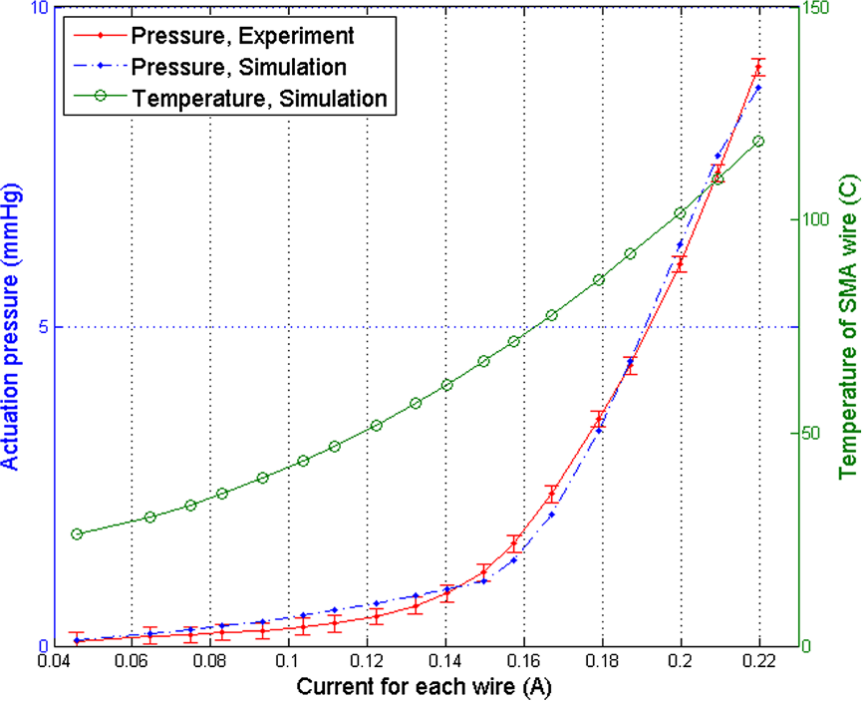
**Comparison of the experimental results and the calculation for the change of pressure by different applied electrical currents.**

Figure 6 compares simulated (blue line) and experimental (red line) results. The estimated stabilised temperature of the wires for each current is also indicated (green line). This figure shows that after activation of the SMA wires, the ACB increased the applied pressure up to 9.06 *mmHg*. This result was achieved by providing 219.88 *mA*. As it can be seen, there is a good agreement between the simulation and experimental results for the entire range of the applied electrical current. The maximum difference between experimentally measured and calculated pressure inside the CM chamber was 0.32 *mmHg*. The average of the calculated austenite start temperature, , was 64°C. According to the simulation results, for currents above 149.7 *mA*, the temperature of the SMA wires was higher than  and thus, the austenite transformation occurred. This transformation is also shown in Figure 6, where a change of slope can be observed around 0.15 *A*. It is worth noting that the high temperature of the SMA wires was not a concern for the performed study as the Lexan layer shielded the CM.

## Conclusion

In this paper an ambulatory active compression bandage using shape memory alloys (SMA) was presented as proof of concept to potentially prevent and eliminate orthostatic intolerance. Based on the obtained experimental results, the prototyped ACB showed a desirable capability of applying initial mechanical pressure similar to commercial compression stockings, and also active alternations in pressure by electrical stimulation. The ACB was able to generate a significant gradient of pressure. In fact, 9.06 *mmHg* was reached when an electrical current of 219.88 *mA* was applied.

The analytical model used in this study could predict changes in pressure generated by the ACB with less than a 0.32 *mmHg* difference with experimental results. The model could be used for improving the ACB design and performing future parametric studies.

Future research includes identifying, via a clinical study, the optimum ACB activation timing to treat disorders associated with excessive swelling of the lower extremities given the maximum variation of pressure that the ACB can achieve. Future research should also investigate reliability of the ACB when it undergoes a large number of cyclic loads.
